# Supplementary material for: Are carbon emissions trading and green financial instruments synergistic? -Comprehensive quantitative research based on content analysis
Source: PLoS One. 2024 Mar 7;19(3):e0298601. doi: 10.1371/journal.pone.0298601 (PMC10919601; doi:10.1371/journal.pone.0298601)
Supplement: S1 Data — (ZIP) [file pone.0298601.s001.zip › Data/地级市Z_F工作报告.zip!/║ú─╧/║ú┐┌╩╨/└┤╫╘╣1⁄2╓┌║┼í╛┬φ┐╦╩2╛▌═°í┐_2011.docx]

各位代表：

　　现在，我代表国务院，向大会作政府工作报告，请各位代表审议，并请全国政协委员提出意见。

　　一、“十一五”时期国民经济和社会发展的回顾

　　“十一五”时期是我国发展进程中极不平凡的五年。面对国内外复杂形势和一系列重大风险挑战，中国共产党团结带领全国各族人民，全面推进改革开放和现代化建设，国家面貌发生了历史性变化。

　　——这五年，我国社会生产力、综合国力显著提高。我们有效应对国际金融危机冲击，保持经济平稳较快发展，胜利完成“十一五”规划的主要目标和任务，国民经济迈上新的台阶。国内生产总值达到39.8万亿元，年均增长11.2%，财政收入从3.16万亿元增加到8.31万亿元。载人航天、探月工程、超级计算机等前沿科技实现重大突破。国防和军队现代化建设取得重大成就。

　　——这五年，各项社会事业加快发展、人民生活明显改善。教育、科技、文化、卫生、体育事业全面进步。城镇新增就业5771万人，转移农业劳动力4500万人;城镇居民人均可支配收入和农村居民人均纯收入年均分别实际增长9.7%和8.9%;覆盖城乡的社会保障体系逐步健全。

　　——这五年，改革开放取得重大进展。重点领域和关键环节改革实现新突破，社会主义市场经济体制更加完善。去年对外贸易总额达到2.97万亿美元，开放型经济水平快速提升。

　　——这五年，我国国际地位和影响力显著提高。我们在国际事务中发挥重要的建设性作用，有力维护国家主权、安全和发展利益，全方位外交取得重大进展。我们成功举办北京奥运会、上海世博会，实现了中华民族的百年梦想。

　　这些辉煌成就，充分显示了中国特色社会主义的优越性，展现了改革开放的伟大力量，极大增强了全国各族人民的自信心和自豪感，增强了中华民族的凝聚力和向心力，必将激励我们在新的历史征程上奋勇前进。

　　五年来，我们主要做了以下工作：

　　(一)加强和改善宏观调控，促进经济平稳较快发展。我们注重把握宏观调控的方向、重点和力度，牢牢掌握经济工作的主动权。“十一五”前期，针对投资增长过快、贸易顺差过大、流动性过剩，以及结构性、输入性物价上涨等问题，采取正确的政策措施，有效防止了苗头性问题演变成趋势性问题、局部性问题演变成全局性问题。近两年，面对百年罕见的国际金融危机冲击，我们沉着应对，科学决策，果断实行积极的财政政策和适度宽松的货币政策。坚持实施一揽子计划，大规模增加政府支出和实行结构性减税，大范围实施重点产业调整振兴规划，大力推进自主创新和加强科技支撑，大幅度提高社会保障水平。坚持扩大内需的战略方针，采取鼓励消费的一系列政策措施，增加城乡居民特别是低收入群众收入，消费规模持续扩大，结构不断升级。实施两年新增4万亿元的投资计划，其中，新增中央投资1.18万亿元。保障性安居工程、农村民生工程和社会事业投资占43.7%，自主创新、结构调整、节能减排和生态建设占15.3%，重大基础设施建设占23.6%，灾后恢复重建占14.8%。政府投资引导带动社会投资，国内需求大幅增加，有效弥补外需缺口，较短时间内扭转经济增速下滑趋势，在世界率先实现回升向好，既战胜了特殊困难、有力地保障和改善了民生，又为长远发展奠定了坚实基础。

　　(二)毫不放松地做好“三农”工作，巩固和加强农业基础。中央财政“三农”投入累计近3万亿元，年均增幅超过23%。彻底取消农业税和各种收费，结束了农民种田交税的历史，每年减轻农民负担超过1335亿元。建立种粮农民补贴制度和主产区利益补偿机制，农民的生产补贴资金去年达到1226亿元。对重点粮食品种实行最低收购价和临时收储政策，小麦、稻谷最低收购价提高了25%到40%。严格保护耕地。着力推进农业科技进步。粮食产量屡创历史新高，去年达到54641万吨，连续7年增产;农民人均纯收入达到5919元，实现持续较快增长。农村综合改革稳步推进，集体林权制度改革、国有农场管理体制改革全面推开。农业农村基础设施加快建设，完成7356座大中型和重点小型水库除险加固，解决2.15亿农村人口饮水安全问题，农民的日子越过越好，农村发展进入一个新时代。

　　(三)大力推进经济结构调整，提高经济增长质量和效益。一是加快产业结构调整和自主创新。积极推进企业技术改造和兼并重组，工业特别是装备制造业总体水平和竞争力明显提高。战略性新兴产业迅速成长。加快建设国家创新体系，实施知识创新工程和技术创新工程，突破了一批产业发展急需的前沿技术、核心技术和关键装备技术，一大批科研成果实现了产业化。服务业快速发展，在国内生产总值中占比提高2.5个百分点。基础设施建设明显加快，五年建成铁路新线1.6万公里，新增公路63.9万公里，其中高速公路3.3万公里，新建、改扩建机场33个，新建和加固堤防1.7万公里。二是扎实推进节能减排、生态建设和环境保护。提出到2020年我国控制温室气体排放行动目标和政策措施，制定实施节能减排综合性工作方案。大力发展清洁能源，新增发电装机容量4.45亿千瓦，其中水电9601万千瓦、核电384万千瓦。关停小火电机组7210万千瓦，淘汰了一批落后的煤炭、钢铁、水泥、焦炭产能。推进林业重点生态工程建设，完成造林2529万公顷。综合治理水土流失面积23万平方公里，加强重点流域水污染防治、大气污染防治和工业“三废”治理。大力发展循环经济。五年累计，单位国内生产总值能耗下降19.1%，化学需氧量、二氧化硫排放量分别下降12.45%、14.29%。三是促进区域经济协调发展。落实区域发展总体战略，颁布实施全国主体功能区规划，制定西部大开发新十年指导意见和一系列区域发展规划，推出促进西藏和四省藏区、新疆等民族地区跨越式发展的新举措。中西部和东北地区发展加快，经济增速等主要指标超过全国平均水平;东部地区经济结构不断优化，自主创新和竞争力逐步提高;地区间基本公共服务差距趋于缩小，各具特色的区域发展格局初步形成。

　　(四)坚定不移深化改革开放，增强经济社会发展内在活力。财政转移支付制度逐步完善，县级基本财力保障机制初步建立。增值税转型全面实施，成品油价格和税费改革顺利推进，资源税改革启动试点，内外资企业税制全面统一。国有大型商业银行股份制改革顺利完成，政策性金融机构改革、农村信用社改革积极推进;平稳解决上市公司股权分置问题，创业板、股指期货和融资融券顺利推出，债券市场稳步发展;深入推进保险业改革开放;人民币汇率形成机制改革有序推进，跨境贸易人民币结算试点不断扩大。国有企业公司制股份制改革、国有资产监管体制改革取得积极进展。邮政体制改革加快推进。制定实施促进中小企业发展和民间投资的一系列政策，非公有制经济发展环境不断改善，多种所有制经济共同发展。

　　不断拓展对外开放的广度和深度。进出口总额年均增长15.9%，结构不断优化。贸易顺差连续两年下降，2010年比上年减少6.4%。利用外资水平进一步提高。企业“走出去”步伐明显加快，累计对外直接投资2200亿美元，对外工程承包和劳务合作营业额3352亿美元。积极参与全球经济治理机制改革和区域合作机制建设，多边、双边经贸合作继续深化。对外援助规模持续扩大。对外开放有力促进了经济发展和结构调整，增加了就业，吸收了先进技术和管理经验，大大提高了我国的国际地位。

　　(五)加快发展社会事业，切实保障和改善民生。始终坚持经济发展与社会发展相协调，围绕改善民生谋发展。把就业放在经济社会发展的优先位置。加强职业培训和就业服务，促进高校毕业生、农村转移劳动力、城镇就业困难人员就业，做好退役军人就业安置工作。实施劳动合同法和就业促进法，普遍提高最低工资标准，推动建立和谐劳动关系。覆盖城乡的社会保障体系建设取得突破性进展，城镇职工基本养老保险实现省级统筹，实施养老保险关系跨省转移接续办法，连续7年提高企业退休人员基本养老金水平，年均增长10%，新型农村社会养老保险试点覆盖24%的县。积极稳妥推进医药卫生体制改革，全面建立城镇居民基本医疗保险制度、新型农村合作医疗制度，惠及12.67亿城乡居民。最低生活保障制度实现全覆盖，城乡社会救助体系基本建立，社会福利、优抚安置、慈善和残疾人事业取得新进展。全国社会保障基金积累7810亿元，比五年前增加5800多亿元。大力实施保障性住房建设和棚户区改造，使1100万户困难家庭住上了新房。我们要持之以恒，努力让全体人民老有所养、病有所医、住有所居。

　　制定和实施国家中长期教育改革和发展规划纲要。五年全国财政教育支出累计4.45万亿元，年均增长22.4%。全面实现城乡免费义务教育，所有适龄儿童都能“不花钱、有学上”。义务教育阶段教师绩效工资制度全面实施。中等职业教育对农村经济困难家庭、城市低收入家庭和涉农专业的学生实行免费。加快实施国家助学制度，财政投入从2006年的18亿元增加到2010年的306亿元，覆盖面从高等学校扩大到中等职业学校和普通高中，共资助学生2130万名，还为1200多万名义务教育寄宿生提供生活补助。加快农村中小学危房改造和职业教育基础设施建设。全面提高高等教育质量和水平，增强高校创新能力。制定并实施国家中长期科学和技术发展规划纲要，中央财政科技投入6197亿元，年均增长22.7%，取得了一系列重大成果。大力加强基层医疗卫生服务能力建设。国家财政安排专项资金，改造和新建2.3万所乡镇卫生院、1500所县医院、500所县中医院和1000所县妇幼保健院，建立了2400所社区卫生服务中心。制定并实施国家中长期人才发展规划纲要。人口规划目标顺利实现。文化体制改革取得重要进展。公共文化服务体系建设明显加快，文化产业蓬勃发展。哲学社会科学和新闻出版、广播影视、文学艺术繁荣进步。城乡公共体育设施建设加快，全民健身活动蔚然成风。法制建设全面推进，“五五”普法顺利完成。创新和加强社会管理，社会保持和谐稳定。

　　抗击汶川特大地震等严重自然灾害的斗争取得重大胜利，汶川灾后恢复重建三年任务两年基本完成，玉树强烈地震和舟曲特大山洪泥石流灾后恢复重建有序推进。经过灾难的洗礼，中国人民更加成熟、自信、坚强，中华民族百折不挠、自强不息的伟大精神不可战胜。

　　五年来，我们不断深化行政管理体制改革，加快转变政府职能，全面完成了新一轮政府机构改革，深入推进依法行政，建设法治政府和服务型政府，推进政务公开，加强行政问责，坚持不懈地开展反腐败斗争，政府自身建设取得积极进展。

　　过去五年，我们是一步一个脚印走过来的，中国人民有理由为此感到自豪!五年的成绩来之不易。这是以胡锦涛同志为总书记的党中央总揽全局、正确领导的结果，是全党全国各族人民共同努力奋斗的结果。在这里，我代表国务院，向全国各族人民，向各民主党派、各人民团体和各界人士，表示诚挚感谢!向香港特别行政区同胞、澳门特别行政区同胞、台湾同胞和海外侨胞，表示诚挚感谢!向关心和支持中国现代化建设的各国政府、国际组织和各国朋友，表示诚挚感谢!

　　我们清醒地认识到，我国发展中不平衡、不协调、不可持续的问题依然突出。主要是：经济增长的资源环境约束强化，投资与消费关系失衡，收入分配差距较大，科技创新能力不强，产业结构不合理，农业基础仍然薄弱，城乡区域发展不协调，就业总量压力和结构性矛盾并存，制约科学发展的体制机制障碍依然较多;服务业增加值和就业比重、研究与试验发展经费支出占国内生产总值比重没有完成“十一五”规划目标。一些群众反映强烈的问题没有根本解决，主要是：优质教育、医疗资源总量不足、分布不均;物价上涨压力加大，部分城市房价涨幅过高;违法征地拆迁等引发的社会矛盾增多;食品安全问题比较突出;一些领域腐败现象严重。我们一定要以对国家和人民高度负责的精神，通过艰苦细致的工作和坚持不懈的努力，加快解决这些问题，让人民满意!

　　回顾“十一五”时期的政府工作，我们进一步加深了以下几个方面的认识和体会。

　　一是必须坚持科学发展。我们战胜各种严峻挑战，靠的是发展;各领域取得的一切成就和进步，靠的是发展;解决前进道路上的困难和问题，仍然要靠发展。我国仍处于并将长期处于社会主义初级阶段，必须坚持以经济建设为中心，坚持科学发展。要以人为本，把保障和改善民生作为一切工作的出发点和落脚点，坚定不移走共同富裕道路，使发展成果惠及全体人民;坚持统筹兼顾，促进城乡、区域、经济社会协调发展;加快转变经济发展方式，大力推进自主创新，节约资源和保护环境，使经济社会发展与人口资源环境相协调，提高发展的全面性、协调性和可持续性。

　　二是必须坚持政府调控与市场机制有机统一。健全的市场机制，有效的宏观调控，都是社会主义市场经济体制不可或缺的重要组成部分。市场作用多一些还是政府作用多一些，必须相机抉择。在应对国际金融危机冲击中，我们加强和改善宏观调控，及时纠正市场扭曲，弥补市场失灵，防止经济出现大的起落，实践证明是完全正确的。我们必须不断完善社会主义市场经济体制，充分发挥市场在资源配置中的基础性作用，激发经济的内在活力，同时，科学运用宏观调控手段，促进经济长期平稳较快发展。

　　三是必须坚持统筹国内国际两个大局。在经济全球化深入发展和对外开放不断深化的条件下，我国经济同世界经济的联系日益紧密，互动和依存不断增强。必须树立世界眼光，加强战略思维，善于从国际形势发展变化中充分把握发展机遇，稳妥应对风险挑战，利用好国内国际两个市场、两种资源，统筹处理好国内发展与对外开放关系，真正做到内外兼顾、均衡发展。

　　四是必须坚持把改革开放作为经济社会发展的根本动力。改革开放是实现国家强盛、人民幸福的必由之路，必须贯穿社会主义现代化建设全过程。我们要以更大的决心和勇气推进改革，提高改革决策的科学性，增强改革措施的协调性，全面推进经济、政治、文化、社会等各方面改革创新，从根本上破除体制机制障碍，最大限度解放和发展生产力，促进社会公平正义。要坚持把改善人民生活作为正确处理改革发展稳定关系的结合点，把改革的力度、发展的速度和社会可承受的程度统一起来，以改革促进和谐稳定，确保人民安居乐业、社会安宁有序、国家长治久安。

　　二、“十二五”时期的主要目标和任务

　　根据《中共中央关于制定国民经济和社会发展第十二个五年规划的建议》，我们编制了《国民经济和社会发展第十二个五年规划纲要(草案)》，提交大会审议。

　　“十二五”是全面建设小康社会的关键时期，是深化改革开放、加快转变经济发展方式的攻坚时期。从国际看，世界多极化、经济全球化深入发展，和平、发展、合作仍是时代潮流。国际金融危机影响深远，世界经济结构加快调整，全球经济治理机制深刻变革，科技创新和产业转型孕育突破，发展中国家特别是新兴市场国家整体实力步入上升期。从国内看，我国发展的有利条件和长期向好的趋势没有改变，工业化、信息化、城镇化、市场化、国际化深入发展，市场需求潜力巨大，资金供给充裕，科技和教育水平整体提升，劳动力素质提高，基础设施日益完善，政府宏观调控和应对重大挑战的能力明显增强，社会大局保持稳定。综合判断国际国内形势，我国发展仍处于可以大有作为的重要战略机遇期。

　　我们要高举中国特色社会主义伟大旗帜，以邓小平理论和“三个代表”重要思想为指导，深入贯彻落实科学发展观，适应国内外形势新变化，顺应各族人民过上更好生活新期待，以科学发展为主题，以加快转变经济发展方式为主线，深化改革开放，保障和改善民生，巩固和扩大应对国际金融危机冲击成果，促进经济长期平稳较快发展和社会和谐稳定，为全面建成小康社会打下具有决定性意义的基础。

　　——我们要推动经济发展再上新台阶。今后五年，我国经济增长预期目标是在明显提高质量和效益的基础上年均增长7%。按2010年价格计算，2015年国内生产总值将超过55万亿元。要继续加强和改善宏观调控，保持价格总水平基本稳定，把短期调控政策和长期发展政策结合起来，坚持实施扩大内需战略，充分挖掘我国内需的巨大潜力，加快形成消费、投资、出口协调拉动经济增长的新局面。

　　——我们要加快转变经济发展方式和调整经济结构。坚持走中国特色新型工业化道路，推动信息化和工业化深度融合，改造提升制造业，培育发展战略性新兴产业。加快发展服务业，服务业增加值在国内生产总值中的比重提高4个百分点。积极稳妥推进城镇化，城镇化率从47.5%提高到51.5%，完善城市化布局和形态，不断提升城镇化的质量和水平。继续加强基础设施建设，进一步夯实经济社会发展基础。大力发展现代农业，加快社会主义新农村建设。深入实施区域发展总体战略和主体功能区战略，逐步实现基本公共服务均等化。促进城乡、区域良性互动，一二三产业协调发展。

　　——我们要大力发展社会事业。坚持优先发展教育，稳步提升全民受教育程度。坚持自主创新、重点跨越、支撑发展、引领未来的方针，完善科技创新体系和支持政策，着力推进重大科学技术突破。研究与试验发展经费支出占国内生产总值比重达到2.2%，促进科技成果更好地转化为生产力。适应现代化建设需要，加强人才培养，努力造就规模宏大的高素质人才队伍。大力加强文化建设，推动文化改革发展实现新跨越，满足人民群众不断增长的精神文化需求。大力发展体育事业。进一步深化医药卫生体制改革，健全基本医疗卫生制度，加快实现人人享有基本医疗卫生服务的目标。创新社会管理体制机制，加强社会管理法律、体制、能力建设，确保社会既充满活力又和谐稳定。

　　——我们要扎实推进资源节约和环境保护。积极应对气候变化。加强资源节约和管理，提高资源保障能力，加大耕地保护、环境保护力度，加强生态建设和防灾减灾体系建设，全面增强可持续发展能力。非化石能源占一次能源消费比重提高到11.4%，单位国内生产总值能耗和二氧化碳排放分别降低16%和17%，主要污染物排放总量减少8%至10%，森林蓄积量增加6亿立方米，森林覆盖率达到21.66%。切实加强水利基础设施建设，推进大江大河重要支流、湖泊和中小河流治理，明显提高基本农田灌溉、水资源有效利用水平和防洪能力。

　　——我们要全面改善人民生活。坚持把增加就业作为经济社会发展的优先目标，为全体劳动者创造公平的就业机会，五年城镇新增就业4500万人。坚持和完善按劳分配为主体、多种分配方式并存的分配制度，努力实现居民收入增长和经济发展同步、劳动报酬增长和劳动生产率提高同步，逐步提高居民收入在国民收入分配中的比重，提高劳动报酬在初次分配中的比重，加快形成合理的收入分配格局。城镇居民人均可支配收入和农村居民人均纯收入年均实际增长超过7%。提高扶贫标准，减少贫困人口。加快完善社会保障制度，进一步提高保障水平。城乡基本养老、基本医疗保障制度实现全覆盖，提高并稳定城乡三项基本医疗保险参保率，政策范围内的医保基金支付水平提高到70%以上，全国城镇保障性住房覆盖面达到20%左右。坚持计划生育基本国策，逐步完善政策，促进人口长期均衡发展，人均预期寿命提高1岁，达到74.5岁。

　　——我们要全面深化改革开放。更加重视改革顶层设计和总体规划，大力推进经济体制改革，积极稳妥地推进政治体制改革，加快推进文化体制、社会体制改革，不断完善社会主义市场经济体制，扩大社会主义民主，完善社会主义法制，使上层建筑更加适应经济基础发展变化，为科学发展提供有力保障。坚持和完善基本经济制度，营造各种所有制经济依法平等使用生产要素、公平参与市场竞争、同等受到法律保护的体制环境。加快财税金融体制改革，积极构建有利于转变经济发展方式的财税体制，构建组织多元、服务高效、监管审慎、风险可控的金融体系。深化资源性产品价格和环保收费改革，建立健全能够灵活反映市场供求关系、资源稀缺程度和环境损害成本的资源性产品价格形成机制。实施更加积极主动的开放战略，培育参与国际合作与竞争新优势，进一步形成互利共赢的开放新格局。

　　——我们要不断加强政府自身改革建设。政府的一切权力都是人民赋予的，必须对人民负责，为人民谋利益，接受人民监督;必须最广泛地动员和组织人民依法管理国家和社会事务，管理经济和文化事业;必须坚持依法治国基本方略，加强维护群众利益的法制建设，推进依法行政;必须实行科学、民主决策，建立健全决策、执行、监督既相互制约又相互协调的运行机制，确保权力正确行使;必须从制度上改变权力过分集中而又得不到制约的状况，坚决惩治和预防腐败;必须保障人民的民主权利和合法权益，维护社会公平正义。

　　总之，经过未来五年努力，实现“十二五”规划的各项目标，我国的综合国力就会有更大的提升，人民生活就会有更大的改善，国家面貌就会发生更大的变化。

　　三、2011年的工作

　　2011年，是“十二五”开局之年，做好今年的工作对于完成“十二五”各项目标任务至关重要。过去一年，我们的各项工作取得了很大成绩。国内生产总值增长10.3%，居民消费价格涨幅控制在3.3%，城镇新增就业1168万人，国际收支状况有所改善。这为做好今年的工作打下了良好基础。

　　今年，我国发展面临的形势仍然极其复杂。世界经济将继续缓慢复苏，但复苏的基础不牢。发达经济体经济增长乏力，失业率居高难下，一些国家主权债务危机隐患仍未消除，主要发达经济体进一步推行宽松货币政策，全球流动性大量增加，国际大宗商品价格和主要货币汇率加剧波动，新兴市场资产泡沫和通胀压力加大，保护主义继续升温，国际市场竞争更加激烈，不稳定不确定因素仍然较多。我国经济运行中一些长期问题和短期问题相互交织，体制性矛盾和结构性问题叠加在一起，加大了宏观调控难度。我们要准确判断形势，保持清醒头脑，增强忧患意识，做好应对风险的准备。

　　今年国民经济和社会发展的主要预期目标是：国内生产总值增长8%左右;经济结构进一步优化;居民消费价格总水平涨幅控制在4%左右;城镇新增就业900万人以上，城镇登记失业率控制在4.6%以内;国际收支状况继续改善。总的考虑是，为转变经济发展方式创造良好环境，引导各方面把工作着力点放在加快经济结构调整、提高发展质量和效益上，放在增加就业、改善民生、促进社会和谐上。

　　实现上述目标，要保持宏观经济政策的连续性、稳定性，提高针对性、灵活性、有效性，处理好保持经济平稳较快发展、调整经济结构、管理通胀预期的关系，更加注重稳定物价总水平，防止经济出现大的波动。

　　继续实施积极的财政政策。保持适当的财政赤字和国债规模。今年拟安排财政赤字9000亿元，其中中央财政赤字7000亿元，继续代地方发债2000亿元并纳入地方预算，赤字规模比上年预算减少1500亿元，赤字率下降到2%左右。要着力优化财政支出结构，增加“三农”、欠发达地区、民生、社会事业、结构调整、科技创新等重点支出;压缩一般性支出，严格控制党政机关办公楼等楼堂馆所建设，出国(境)经费、车辆购置及运行费、公务接待费等支出原则上零增长，切实降低行政成本。继续实行结构性减税。依法加强税收征管。对地方政府性债务进行全面审计，实施全口径监管，研究建立规范的地方政府举债融资机制。

　　实施稳健的货币政策。保持合理的社会融资规模，广义货币增长目标为16%。健全宏观审慎政策框架，综合运用价格和数量工具，提高货币政策有效性。提高直接融资比重，发挥好股票、债券、产业基金等融资工具的作用，更好地满足多样化投融资需求。着力优化信贷结构，引导商业银行加大对重点领域和薄弱环节的信贷支持，严格控制对“两高”行业和产能过剩行业贷款。进一步完善人民币汇率形成机制。密切监控跨境资本流动，防范“热钱”流入。加强储备资产的投资和风险管理，提高投资收益。

　　今年，重点要做好以下几方面工作。

　　(一)保持物价总水平基本稳定

　　当前，物价上涨较快，通胀预期增强，这个问题涉及民生、关系全局、影响稳定。要把稳定物价总水平作为宏观调控的首要任务，充分发挥我国主要工业品总体供大于求、粮食库存充裕、外汇储备较多等有利条件，努力消除输入性、结构性通胀因素的不利影响，消化要素成本上涨压力，正确引导市场预期，坚决抑制价格上涨势头。要以经济和法律手段为主，辅之以必要的行政手段，全面加强价格调控和监管。一是有效管理市场流动性，控制物价过快上涨的货币条件。把握好政府管理商品和服务价格的调整时机、节奏和力度。二是大力发展生产，保障主要农产品、基本生活必需品、重要生产资料的生产和供应。落实“米袋子”省长负责制和“菜篮子”市长负责制。三是加强农产品流通体系建设，积极开展“农超对接”，畅通鲜活农产品运输“绿色通道”。完善重要商品储备制度和主要农产品临时收储制度，把握好国家储备吞吐调控时机，搞好进出口调节，增强市场调控能力。四是加强价格监管，维护市场秩序。特别要强化价格执法，严肃查处恶意炒作、串通涨价、哄抬价格等不法行为。五是完善补贴制度，建立健全社会救助和保障标准与物价上涨挂钩的联动机制，绝不能让物价上涨影响低收入群众的正常生活。
